# The Effectiveness of the Surgical Correction of Vesicoureteral Reflux on Febrile Urinary Tract Infections after a Kidney Transplant: A Single-Center Retrospective Study

**DOI:** 10.3390/jcm13175295

**Published:** 2024-09-06

**Authors:** Andre E. Varaschin, Gabriella G. Gomar, Amanda M. Rocco, Silvia R. Hokazono, Quelen I. Garlet, Cláudia S. Oliveira

**Affiliations:** 1 Instituto de Pesquisa Pele Pequeno Principe, Curitiba 80250-060, PR, Brazil; andrevaraschin@gmail.com; 2 Faculdades Pequeno Principe, Curitiba 80230-020, PR, Brazil; gabriella.gomar@aluno.fpp.edu.br; 3 Programa de Residência Médica em Urologia, Hospital Universitário Cajuru, Curitiba 80050-050, PR, Brazil; amandarocco96@gmail.com (A.M.R.); silvia.hokazono@gmail.com (S.R.H.); 4 Programa de Pós-graduação em Farmacologia, Universidade Federal do Paraná, Curitiba 81531-980, PR, Brazil

**Keywords:** ureteral reimplantation, post-transplant, surgical correction, vesicoureteral reflux

## Abstract

**Background/Objectives**: Vesicoureteral reflux (VUR) is considered one of the major causes of post-renal transplant febrile urinary tract infections (UTI), leading to impaired renal function and the premature loss of the renal graft. We aimed to evaluate whether surgical VUR correction, such as open redo ureteric reimplantation, could be an option for treatment and provide better outcomes in post-transplant care for patients with UTI compared to their pre-VUR correction clinical state. **Methods**: Our study presents a retrospective analysis of 10 kidney transplant recipients with febrile UTI at the Renal Transplant Service of a Brazilian public hospital from 2010 to 2020. We selected patients who primarily underwent a surgical correction of post-transplant VUR, which was corrected by extravesical reimplantation without a stent in all patients by the same professional surgeon. **Results**: From 710 patients who received kidney transplants, 10 patients (1.4%) suffered from febrile UTI post-transplant and underwent surgical correction for VUR. Despite the study’s limitations, such as its retrospective nature and limited sample size, the efficacy of open extravesical ureteral reimplantation in reducing post-operative febrile UTI in renal transplant patients was observed. **Conclusions**: As febrile UTI can contribute significantly to patient mortality after kidney transplantation and VUR emerges as a major cause of post-transplant febrile UTI, it is essential to treat it and consider the surgical outcome. This study emphasizes the timely detection and effective treatment of VUR via extravesical techniques to reduce febrile UTI occurrences post-transplant and it contributes insights into the role of surgical interventions in addressing VUR-related complications post-kidney transplantation.

## 1. Introduction

Kidney transplantation stands as a life-changing medical procedure, offering a renewed lease on life for individuals suffering from end-stage renal disease [1]. While the success of kidney transplantation has brought about significant improvements in the quality of life for many recipients [2], certain complications can arise, casting shadows on the post-transplant journey. Following kidney transplantation, urinary tract infections (UTI) are one of the most related factors in graft loss [3] and patient mortality [4]. UTI incidence varies from 35 to 60% [5], presenting a complex challenge for both transplant recipients and healthcare providers. In this context, vesicoureteral reflux (VUR) is considered one of the major causes of post-renal transplant febrile UTI, leading to impaired renal function and the premature loss of the renal graft [6]. The surgical correction of VUR, whether open or endoscopic, is necessary for a large number of patients. Open surgical techniques include pyelo-ureteric or uretero-ureteric anastomosis and ureteric reimplantation [7]. Open surgery for VUR correction in transplant patients is the approach that yields the best results [7,8]. VUR is defined as an abnormal flow of urine from the bladder back into the ureters that potentially reaches the kidneys [9]. This condition becomes particularly significant in the post-kidney transplant scenario, as it has been identified as one of the major contributors to UTI in transplant recipients, occurring in approximately 86% of the patients with febrile UTI [10,11]. This abnormal condition of the urinary tract is a risk factor for renal damage, both in the non-transplanted population and the immunosuppressed transplanted population, as demonstrated in retrospective observational studies [11,12]. VUR is a risk factor for UTI because of the possible contamination when the urine refluxes back to the ureters and kidneys. This scenario allows the bacteria colonizing the bladder to be transported to the upper urinary tract [13]. Additionally, general urinary stasis and previous urinary lesions can also increase the incidence of UTI [14]. An approach applied to reduce recurrent UTI after renal transplantation is antibiotic prophylaxis [11]. However, some authors report that the prolonged use of this resource may result in the emergence of multi-resistant bacteria and an increase in plasma creatinine levels [11]. Therefore, it is essential to analyze the incidence of febrile and lower UTI in patients who experience VUR following renal transplantation, as they are more susceptible to infection recurrence. In this article, we evaluate the relationship between UTI after kidney transplantation and VUR surgical correction in a Brazilian hospital. Here, we report our retrospective experience with 10 renal-transplanted patients suffering from recurrent febrile UTI. These patients had undergone an open surgical correction of post-transplant VUR using the extravesical ureteric reimplantation technique as an option to improve their clinical outcomes compared to the pre-VUR correction clinical state.

## 2. Materials and Methods

### 2.1. Patients and Methods

A retrospective analysis was carried out on the medical records of all patients undergoing kidney transplantation at the Renal Transplant Service of a public hospital, from 2010 to 2020, located in Curitiba city, Paraná state, Brazil. From those patients, we selected the ones who primarily underwent the surgical correction of post-transplant VUR (diagnosed by cystourethrography) due to febrile UTI (diagnosed as >10^5^ CFU/mL on urine culture with fever, allograft pain, chills, malaise, or bacteremia with the same organism in urine). Cystourethrography was performed in all kidney transplant patients during the second episode of febrile UTI or the first febrile UTI with abnormalities on imaging tests (usually ultrasound or computed tomography). All the UTI patients diagnosed with VUR underwent surgical correction. Patients with febrile UTI who did not have a diagnosis of VUR were not selected for this study as it was beyond the scope of our study. This study was approved by the Ethical Committees of the Faculdades Pequeno Príncipe (#4.425.915) and Pontifícia Universidade Católica do Paraná (#4.573.798). The VUR was corrected by extravesical reimplantation without a stent in all patients by the same professional surgeon. The surgical technique was an open surgery, with an incision in the iliac fossa; in the same incision as the previous transplant, the distal ureter was mobilized and dissected up to the ureterovesical junction, creating a path in the detrusor wall while ensuring not to violate the bladder mucosa. The detrusor wall was then approximated over the ureter to create a sufficient tunnel to prevent reflux. Patient characteristics were recorded, including the date of transplant, etiology of end-stage renal disease, sex, age at transplantation, donor classification of transplant, warm and cold ischemia duration, immunosuppression protocol, number of rejection episodes (pre- and post-VUR correction), age at anti-reflux revision surgery, grade of VUR using the International Reflux Study Committee Scale, episodes of febrile UTI (pre- and post-VUR correction), episodes of lower UTI (simple cystitis, i.e., the presence of >10^5^ CFU/mL on urine culture with local urinary symptoms, such as dysuria, frequency, or urgency, but no systemic symptoms, such as fever or allograft pain; pre- and post-VUR correction) and UTI etiologic agents. The intervals between original transplantation, diagnosis of VUR, surgical correction, and the last clinical visit were also recorded. The serum creatinine levels recorded pre- and post-VUR correction surgery were used to calculate the glomerular filtration rate (GFR) using the CKD-EPI formula provided by the Brazilian Society of Nephrology.

### 2.2. Statistical Analysis

Data are presented as mean ± standard deviation (SD) or median ± interquartile range for parametric and non-parametric data, respectively. The adequacy for the Gaussian model was assessed using the Kolmogorov–Smirnov normality test and Levene’s test was performed to ensure homoscedasticity. Repeated measures analysis of pre- and post-intervention factors was performed using Wilcoxon matched-pairs signed-rank test for non-parametric data or mixed effect repeated measures ANOVA using pre- and post-VUR surgical correction as a matching factor. All analyses were performed using the software Excel© version 2408 and GraphPad Prism© version 8.4.2. Statistical significance was considered when *p* < 0.05.

## 3. Results

During this retrospective study, we identified 10 patients (1.41%) diagnosed with post-transplantation VUR and recurrent febrile UTI who underwent primarily open VUR surgical correction (extravesical ureteric reimplantation technique), from a total of 710 patients who had undergone kidney transplantation during the time of the study. VUR correction was performed in two patients with one episode of febrile UTI and who had undergone ultrasound or urotomography suggestive of VUR, and in eight patients with two or more episodes of febrile UTI, regardless of the time since transplantation. All of the patients were confirmed to have had VUR through preoperative cystourethrography. Patients and kidney transplantation characteristics are presented in Table 1.

The majority of the patients were female (80%) with an average age of 35.4 ± 17.3 years old, while for male patients (20%), the average age was 60.0 ± 2.8 years old at the time of kidney transplantation. The end-stage renal etiologies included *Diabetes mellitus* (30%), chronic glomerulonephritis (40%), polycystic kidney disease (10%), nephrosclerosis (10%), and lupus (10%). Ninety percent of the graph type was cadaveric. Regarding the immune suppression protocol, 80% of the patients were treated with PRED + TAC + MIC before VUR correction, and after VUR correction this percentage increased to 90% (Table 2). Before VUR correction, four patients (40%) suffered tissue rejection. After the VUR procedure, only one of these patients did not have rejection episodes, while the remaining three patients continued enduring rejection, and from those, two patients lost the graft. In addition, there was no clinical, laboratory, or ultrasound evidence of ureteral stricture after VUR correction in these 10 patients.

**Figure 1:**
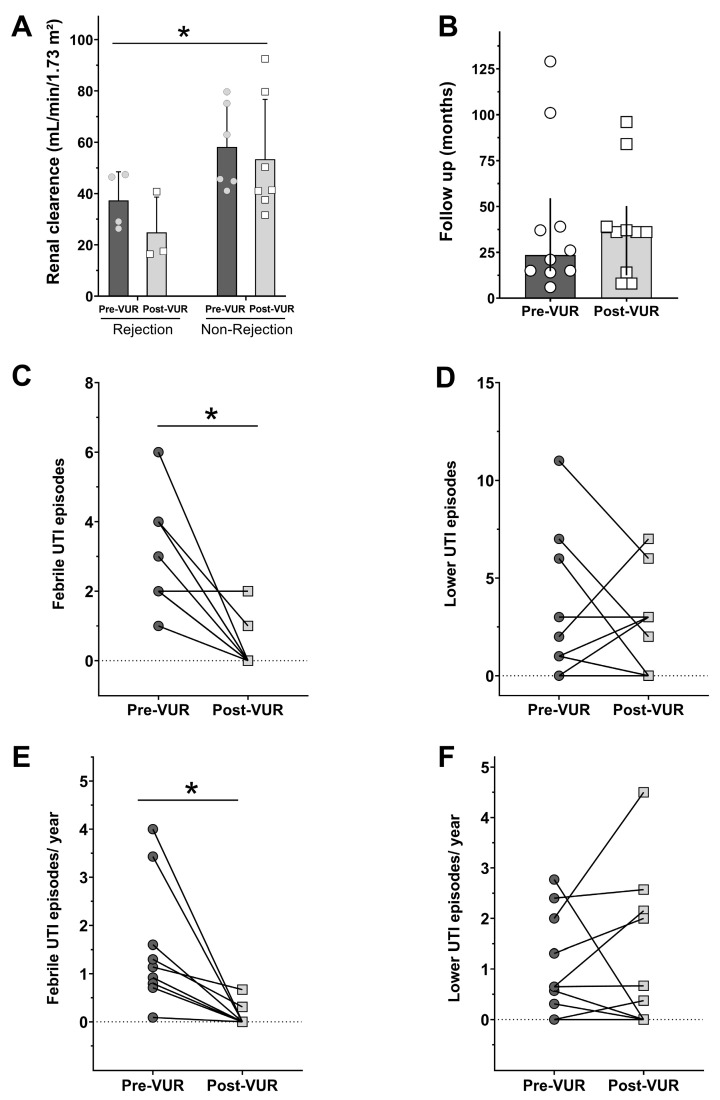
Renal clearance from patients pre- and post-VUR surgical correction that suffered or did not suffer graft rejection (**A**). Time of patient’s follow-up (**B**). Total and per year number of febrile (**C**,**E**) and lower (**D**,**F**) UTI, respectively. * indicates the statistical difference from rejection group (**A**) or from pre-VUR data (**C**,**E**), *p* < 0.05.

Following the VUR correction procedure, we gathered data from renal clearance and febrile and lower UTI episodes and compared them to pre-VUR correction data, considering the follow-up period of the patient (Figure 1). We observed a statistical difference in the GFR between patients who experienced graft rejection from those who did not experience rejection (Figure 1A, F_(1,16)_ = 8.105; *p* = 0.0117), while the VUR correction procedure did not influence renal clearance. Patients’ follow-up time was homogeneous between pre-VUR and post-VUR correction events (Figure 1B). A paired analysis showed a statistical difference in the febrile UTI total number of episodes between pre-VUR and post-VUR surgical correction (Figure 1C, *p* = 0.0039), while lower UTI episodes did not vary after VUR correction (Figure 1D). The same pattern was maintained when we arranged the UTI data by year, as we observed that febrile UTI are more likely to occur before VUR correction (Figure 1E, *p* = 0.002) and the non-febrile UTI annual rate did not differ after the procedure (Figure 1F). The small sample may bias the statistical conclusions; therefore, we tested the power of the analysis and found the following: the power of the performed two-tailed test with alpha = 0.050:0.993. Therefore, the β value is 1 − 0.993 = 0.007 or 0.7%. The β value should be lower than 20% for a sample size to be considered suitable for the analysis.

The pre- and post-VUR correction features regarding UTI information from each patient are shown in Table 3. The patients’ follow-up varied from 6 to 129 months pre-VUR correction, while post-VUR correction follow-up time varied from 8 to 96 months. Meanwhile, the VUR grade varied from II to V among seven patients (70%), as the medical records did not inform the VUR grade for the remaining three patients. The lower UTI in patients pre-VUR correction was observed in eight patients (80%) during the follow-up; three patients experienced 1 episode and the remaining five patients experienced 2 to 11 lower UTI episodes. All lower and upper infections were treated with antibiotics before and after the VUR correction. The treatment duration was typically 7 days for lower UTI and 14 days for febrile UTI. After VUR correction, the number of lower UTI episodes decreased by half; four patients (40%) did not experience lower UTI episodes, while the remaining six patients (60%) experienced two or more lower UTI episodes. Regarding the UTI etiologic agents, *Escherichia coli* was found to be the infection agent in 62.5% of the lower UTI episodes pre-VUR correction. Of those episodes, 30% of the cases were caused by an extended-spectrum beta-lactamase (ESBL) strain and 10% were caused by a multidrug-resistant (MDR) strain. *Klebsiella* sp. was found in 12.5% of the lower UTI episodes, while *Proteus* sp. and *Raoultella* sp. MDR were detected in 3.1% of lower UTI episodes. Meanwhile, the etiologic agent was not identified in 18.75% of the lower UTI episodes pre-VUR correction. On the other hand, lower UTI episodes after VUR correction included the following etiologic agents: *E. coli* (58.33% of the cases, from that 28.57% were ESBL strain and 14.28% were MDR strain), *Klebsiella* sp. (20.83% of the cases, from those 60% were identified as *Klebsiella pneumoniae*), *Morganella morganii* (12.50% of the cases), *Proteus* sp. (4.16% of the cases) and *Citrobacter* sp. (4.16% of the cases).

The episodes of febrile UTI before VUR correction varied from one to six per patient during the follow-up period. Several patients (40%) experienced two episodes of febrile UTI before the anti-reflux surgery. After VUR correction, only two patients experienced febrile UTI episodes. *E. coli* was detected as the etiologic agent in 33.3% of the febrile UTI episodes (from those 44.4% were ESBL strain and 44.4% were MDR strain). *Klebsiella* sp. ESBL and *Enterobacter* sp. were detected as the etiologic agent in 11.1% and 3.7% of the febrile UTI episodes, respectively. The etiologic agent was not identified in 51.85% of the febrile UTI episodes pre-VUR correction. After the VUR correction, the etiologic agent was not identified in one episode and *E. coli* ESBL and *Klebsiella pneumoniae* were detected as etiological agents in the remaining two episodes. Additional information on patients included the fact that two patients underwent hemodialysis due to graft loss. Moreover, over the course of the study, we experienced patient loss, accounting for two deaths due to COVID-19 complications and one death due to neurocryptococcosis.

## 4. Discussion

In our study, we evaluated a total of 710 patients who underwent kidney transplantation, resulting in 10 patients experiencing VUR and febrile UTI outcomes. Here, we analyzed several factors surrounding the 10 patients who underwent an open extravesical technique that involved increasing the length of the submucosal tunnel without the placement of a double J catheter. The follow-up duration varied from 6 to 129 months pre-VUR correction and from 8 to 96 months post-VUR correction. The VUR grade, when reported, ranged from II to IV. Several etiological agents were identified in patients with UTI, including the microorganisms *E. coli*, *Proteus* sp., *Klebsiella* sp., and *Raoultella* sp.

Several factors have been reported to collaborate in the increase in the risk of post-transplant VUR. Such factors can be divided into two main categories: modifiable and non-modifiable risk factors [6]. Non-modifiable factors include recipient characteristics, such as sex, urinary tract abnormalities, neurological disorders, atrophic bladder, *Diabetes mellitus*, and dialysis vintage. Meanwhile, modifiable factors include the surgical technique of ureteral reimplantation and surgical team experience [15]. The tunnel length in the ureteral reimplantation technique in kidney transplantation is one of the most important modifiable factors in the appearance of VUR after kidney transplantation [16]. Accordingly, several authors have proposed that the length of the tunnel in reimplantation plays a role in postoperative VUR [17]. Neuhaus et al. [16] described a submucosal tunnel < 1 cm in all cases of post-transplant VUR. When the tunnel length was increased to a minimum of 3 cm, a 100% resolution in 3–6 months of follow-up was observed. The European Association of Urology Guidelines recommends that the anti-reflux tunnel for ureterovesical anastomosis should be set from 3 to 4 cm [18]. In the Medical Center where this study was carried out, the protocol and technique used for ureteral reimplantation in kidney transplant surgery is the Lich-Gregoir technique. Unfortunately, we do not have information on the length of the submucosal tunnel nor the quality of the bladder wall from our patients undergoing kidney transplantation, as these data were not available in the medical records. 

The possible impact of surgeon experience on the development of post-renal transplant VUR has been evaluated in some studies. Cash et al. [19] investigated the effect of surgeon experience on rates of post-renal transplant complications, and in their study, a significantly higher rate of urological complications was observed due to the inexperience of the surgical team (6.6% versus 2.7%). In our hospital setting, kidney transplant surgery was performed by different surgical teams. However, the surgical procedure used to correct VUR after kidney transplantation was performed by the same professional surgeon.

UTI episodes can occur as asymptomatic bacteriuria that may not require treatment in adult non-pregnant immunocompetent individuals [20]. However, a severe UTI can cause pyelonephritis or septicemia in immunosuppressed post-renal transplant patients. There is a consensus that VUR might increase the risk for UTI; however, the long-term consequences for the graft remain controversial, as reports on the long-term influence of UTI on renal graft function are inconsistent [21,22]. Acute graft pyelonephritis has been reported as an independent risk factor for declining renal function in a study involving 172 recipients [23]. However, other reports do not confirm this relationship [22,24,25]. Although UTI does not directly affect graft survival, they may indirectly affect the graft through bacteremia, episodes of acute rejection, or cytomegalovirus infection. In this context, Coulthard and Keir [26] observed that 40% of transplanted kidneys are at risk of renal scars due to pyelonephritis, and in the presence of UTI and VUR, half of these patients suffered significant renal damage with a decrease in the GFR in patients evaluated by renal scintigraphy.

As mentioned above, the renal graft is particularly sensitive, directly or indirectly, to the consequences of UTI [23]. Improvements in surgical procedures, the rapid removal of the urethral catheter, and antibiotic prophylaxis have reduced the incidence of UTI in the immediate postoperative period [23]. However, the incidence of UTI is higher in transplanted patients compared to the normal population [26]. Abbott et al. [12], in a large retrospective study, revealed that late UTI occurring after renal transplantation (more than six months post-surgery) are associated with a significant increase in the risk of death. However, they could not establish whether UTI were the primary cause of death or whether they were a consequence of an underlying disease. We observed three deaths in our study, and in none of them, the primary cause of death was due to urinary infection.

It is established that the increased incidence of UTI is proportional to the intensity of immunosuppressive therapy [10,12,23,24,25,26,27,28]. Muller et al. [27] reported that transplanted patients with chronic rejection have experienced more UTIs than those without signs of rejection. In contrast, Giral et al. [25] reported that episodes of rejection do not constitute a risk factor for acute pyelonephritis. Pelle et al. [23] found no association between episodes of acute rejection and acute pyelonephritis. Pyelonephritis may be a trigger for the immune response, which increases the risk of acute rejection [23]. Similar occurrences of acute rejection, shortly after acute pyelonephritis, have been reported by other authors [23]. We know that the incidence of acute rejection within the first year is around 7.9%, and inadequate immunosuppression increases the risk of rejection of the renal allograft after the transplantation. In this study, we had four patients (40%) who experienced graft rejection. It is the service’s protocol to reduce immunosuppression dosage in patients with severe infections, including febrile UTI, which may explain the high rejection rate of patients in this study. 

Regarding patients with febrile UTI and associated VUR after kidney transplant surgery, there is no consensus on when and for which patient surgery to correct VUR is recommended. Krishnan et al. [29] consider that once VUR is identified after a significant febrile UTI, surgical correction should be indicated for the patient. The degree of reflux is also an important factor in assessing the transplant results. Favi et al. [21] evaluated post-renal transplant patients with low-grade VUR (grade I-III) and showed that it does not affect long-term renal graft function, concluding that there is no surgical indication for reflux repair in low-grade reflux. On the other hand, available data suggest that symptomatic grade IV and V reflux can lead to progressive graft dysfunction and premature graft loss [6].

The surgical treatment of VUR can be endoscopic or open surgery. Open surgery can be either intravesical or extravesical ureteral reimplantation, uretero-ureterostomy, or pyeloureterostomy. The reference for the treatment of symptomatic VUR is open ureteral reimplantation surgery, with success rates of 83% to 100% [28]. In a series with 60 patients undergoing open VUR correction post-transplantation, the number of UTI decreased significantly after correction, with an average number of febrile UTI before surgery of four and after surgery of one [11]. Krishnan et al. [29], in a series of 20 patients who underwent extravesical ureteral reimplantation, showed an improvement rate of infections of 75%.

Endoscopic correction is a minimally invasive procedure and is gaining popularity. Duty et al. [30] demonstrated that post-transplant VUR patients treated endoscopically in grade I and II VUR had a success rate of 90%, whereas in grade III and IV VUR, the success rate was 31%, concluding that endoscopic management can be performed in patients with low VUR (grade I–II), and transplant patients with grade III and IV reflux or high bladder pressures are best managed with open surgery. Our service has no experience with endoscopic reflux correction techniques. As demonstrated by this study, all of our patients experienced a decrease in the number of infections after VUR correction.

Despite the valuable insights gained from our study, several limitations must be acknowledged, including its retrospective nature, with potential selection bias; a lack of critical information, which could lead to unreliable inferences; and a small sample size, risking a misestimation of success rates and complications. Although the sample size for clinical relevance may be small, the statistical tests applied were reliable as we detected a high statistical power in the analysis. Additionally, there is no large volume of cases in most series because the incidence of febrile ITU followed by surgery for VUR correction is low, as mentioned above. In our study, cysto-urethrography was performed post-VUR correction in only one patient, and unfortunately, we did not perform cysto-urethrography to evaluate the outcome of reflux correction. However, we believe that this procedure is not necessary unless there is persistence of infections, decreased renal function, or underlying lower urinary tract abnormality. Our main goal was to report here our experience with a single professional surgeon performing post-renal transplant surgical correction and analyze the ITU outcome in such patients. Despite some limitations, our findings contribute to the understanding of VUR management in renal transplant recipients.

## 5. Conclusions

Our study sheds light on the incidence, risk factors and management outcomes of VUR in a small sample of renal transplant recipients. We found that VUR is associated with a notable incidence of febrile UTI in this population, with surgical correction being necessary in a small, however, significant percentage of cases. The length of the submucosal tunnel in ureteral reimplantation emerged as a critical factor influencing postoperative VUR, emphasizing the importance of the surgical technique. In conclusion, open surgery for the correction of post-kidney transplantation VUR can decrease the number of recurrent febrile UTI and prevent further kidney damage due to febrile UTI. The extravesical urethral reimplantation method is an effective and safe option for correcting VUR after kidney transplantation. Future research endeavors with larger cohorts and prospective designs are warranted to validate our findings and optimize the management of VUR in renal transplant recipients. 

## Figures and Tables

**Table 1:** Patients and kidney transplantation characteristics.

	All	Female	Male
N (%)	10 (100)	8 (80)	2 (20)
Etiology of chronic renal failure (%)			
Polycystic kidney disease	1 (10)	-	1 (50)
Diabetes mellitus	3 (30)	3 (37.5)	-
Chronic glomerulonephritis	4 (40)	4 (50)	-
Nephrosclerosis	1 (10)	-	1 (50)
Lupus	1 (10)	1 (12.5)	-
Age at the time of kidney transplantation (years [mean ± SD])	40.3 ± 18.5	35.4 ± 17.3	60.0 ± 2.8
Graft type (%)			
Live	1 (10)	1 (12.5)	-
Cadaveric	9 (90)	7 (87.5)	2 (100)
Ischemia duration			
Live			
Warm ischemia (s)	230	230	-
Cold ischemia (min)	75	75	-
Cadaveric			
Cold ischemia (h [mean ± SD])	19.1 ± 5.5	17.7 ± 3.1	22.6 ± 10.4
Age at VTU correction surgery (years [mean ± SD])	43.3 ± 18.2	38.8 ± 17.6	61.0 ± 2.8

**Table 2:** Immunosuppression protocols and rejection rates.

	Number of Patients (%)
Immunosuppressive treatment protocol before VUR correction	
PRED + TAC + SIR	1 (10)
PRED + TAC + AZA	1 (10)
PRED + TAC + MIC	8 (80)
Immunosuppressive treatment protocol after VUR correction	
PRED + TAC + AZA	1 (10)
PRED + TAC + MIC	9 (90)
Rejection before VUR correction (number of episodes)	
0	6 (60)
1	4 (40)
Rejection after VUR correction (number of episodes)	
0	7 (70)
1	3 (30)
Fisher’s test on rejection pre- and post-VUR	
Relative Risk for rejection after VUR correction (95% IC)	0.8077 (0.3483 to 2.101); *p* = 0.99
NNT	9.1

Abbreviations: PRED = prednisone; TAC = tacrolimus; SIR = sirulimus; MIC = micophenolate; AZA = azathioprine; NNT: number needed to treat.

**Table 3:** Pre- and post-VUR surgical correction features related to UTI per patient.

	Pre-VUR Surgical Correction						Post-VUR Surgical Correction					
Patient	Follow-Up (months)	VUR Grade	Lower UTI Episodes	Etiologic Agent	Febrile ITU Episodes	Etiologic Agent	Follow-Up (months)	Lower UTI Episodes	Etiologic Agent	Febrile ITU Episodes	Etiologic Agent	Other Information
01	21	N.I.	1	*Proteus* spp. (1×)	2	*Escherichia coli* (ESBL) (1×); *Klebsiella pneumoniae* (ESBL) (1×)	36	0	-	2	*Escherichia coli* (ESBL) (1×); *Klebsiella pneumoniae* (1×)	Graft rejection pre-VUR correction; Death by neurocryptococcosis
02	14	N.I.	0	-	4	*Escherichia coli* (ESBL) (2×); N.I. (2×)	96	3	*Proteus* spp. (1×); *Klebsiella* sp. (1×); *Citrobacter* sp. (1×)	0	-	-
03	37	II	2	N.I. (2×)	4	*Escherichia coli* (MDR) (2×); N.I. (2×)	39	7	*Klebsiella* sp. (1×); *Escherichia coli* (5×), (MDR) (1×)	1	N.I.	-
04	26	N.I.	6	*Escherichia coli* (ESBL) (5×); *Klebsiella* sp. (1×)	2	N.I. (2×)	84	0	-	0	-	Graft rejection pre-and post-VUR correction; Death by COVID-19
05	15	IV	3	*Klebsiella* sp. (3×)	1	N.I. (1×)	19	3	*Escherichia coli* (3×)	0	-	Death by COVID-19
06	15	III	0	-	2	N.I. (2×)	8	0	-	0	-	Graft rejection pre-and post-VUR correction; Graft loss (transplant glomerulopathy)–started hemodialysis
07	101	IV	11	*Escherichia coli* (7×); (MDR) (1×); N.I. (3×)	6	*Escherichia coli* (MDR) (2×); *Enterobacter* sp. (1×); N.I. (3×)	36	6	*Escherichia coli* (ESBL) (3×); Morganella morganii (3×)	0	-	-
08	6	IV	1	*Escherichia coli* (1×);	2	*Klebsiella* sp. (ESBL) (2×)	8	3	*Klebsiella pneumoniae* (3×)	0	-	Graft rejection pre-and post-VUR correction; Graft loss (rejection) started hemodialysis
09	39	V	1	*Raoultella* sp. (MDR) (1×)	3	*Escherichia coli* (1×); N.I. (2×)	37	0	-	0	-	-
10	129	III (right kidney) ^#^	7	*Escherichia coli* (4×) (ESBL) (1×); (MDR) (1×); N.I. (1×)	1	*Escherichia coli* (ESBL) (1×)	36	2	*Escherichia coli* (ESBL) (1×), (MDR) (1×)	0	-	-

Abbreviations: ESBL = Extended-Spectrum Beta-Lactamase; MDR = multidrug-resistant; N.I. = not informed; ^#^ en block transplant, pediatric donor.

## Data Availability

All data are available in the manuscript, and additional information can be requested from the corresponding author.

## References

[1] Abecassis M., Bartlett S.T., Collins A.J., Davis C.L., Delmonico F.L., Friedewald J.J., Hays R., Howard A., Jones E., Leichtman A.B. (2008). Kidney transplantation as primary therapy for end-stage renal disease: A National Kidney Foundation/Kidney Disease Outcomes Quality Initiative (NKF/KDOQITM) conference. Clin. J. Am. Soc. Nephrol..

[2] Kostro J., Hellmann A., Kobiela J., Skóra I., Lichodziejewska-Niemierko M., Dębska-Ślizień A., Śledziński Z. (2016). Quality of life after kidney transplantation: A prospective study. Transplant. Proc..

[3] Memikoğlu K., Keven K., Şengül S., Soypaçaci Z., Ertürk S., Erbay B. (2007). Urinary tract infections following renal transplantation: A single-center experience. Transplant. Proc..

[4] Chuang P., Parikh C.R., Langone A. (2005). Urinary tract infections after renal transplantation: A retrospective review at two US transplant centers. Clin. Transplant..

[5] Meena P., Bhargava V., Rana D.S., Bhalla A.K. (2021). Urinary tract infection in renal transplant recipient: A clinical comprehensive review. Saudi J. Kidney Dis. Transplant..

[6] Brescacin A., Iesari S., Guzzo S., Alfieri C.M., Darisi R., Perego M., Puliatti C., Ferraresso M., Favi E. (2022). Allograft vesicoureteral reflux after kidney transplantation. Medicina.

[7] Capozza N., Caione P. (2007). Vesicoureteral reflux: Surgical and endoscopic treatment. Pediatr. Nephrol..

[8] Dogan H.S., Bozaci A.C., Ozdemir B., Tonyali S., Tekgul S. (2014). Ureteroneocystostomy in primary vesicoureteral reflux: Critical retrospective analysis of factors affecting the postoperative urinary tract infection rates. Int. Urol.

[9] Khoury A.E., Bägli D.J., Wein A.J., Kavoussi L.R., Novick A.C., Partin A.W., Peters C.A. (2016). Vesicoureteral Reflux. Campbell Walsh Urology.

[10] Mastrosimone S., Pignata G., Maresca M.C., Calconi G., Rabassini A., Butini R., Fandella A., Di Falco G., Chiara G., Caldato C. (1993). Clinical significance of vesicoureteral reflux after kidney transplantation. Clin. Nephrol..

[11] Dinckan A., Aliosmanoglu I., Kocak H., Gunseren F., Mesci A., Ertug Z., Yucel S., Suleymanlar G., Gurkan A. (2013). Surgical correction of vesico-ureteric reflux for recurrent febrile urinary tract infections after kidney transplantation. BJU Int..

[12] Abbott K.C., Swanson S., Richter E.R., Bohen E.M., Agodoa L.Y., Peters T.G., Barbour G., Lipnick R., Cruess D.F. (2004). Late urinary tract infection after renal transplantation in the United States. Am. J. Kidney Dis..

[13] Garcia-Roig M.L., Kirsch A.J. (2016). Urinary tract infection in the setting of vesicoureteral reflux. F1000Research.

[14] Van Batavia J.P., Ahn J.J., Fast A.M., Combs A.J., Glassberg K.I. (2013). Prevalence of urinary tract infection and vesicoureteral reflux in children with lower urinary tract dysfunction. J. Urol..

[15] Sui W., Lipsky M.J., Matulay J.T., Robins D.J., Onyeji I.C., James M.B., Theofanides M.C., Wenske S. (2018). Timing and predictors of early urologic and infectious complications after renal transplant: An analysis of a New York statewide database. Exp. Clin. Transplant..

[16] Neuhaus T.J., Schwöbel M., Schlumpf R., Offner G., Leumann E., Willi U. (1997). Pyelonephritis and vesicoureteral reflux after renal transplantation in young children. J. Urol..

[17] Yucel S., Akin Y., Celik O., Erdogru T., Baykara M. (2010). Endoscopic vesicoureteral reflux correction in transplanted kidneys: Does injection technique matter?. J. Endourol..

[18] EAU Guidelines Presented at the EAU Annual Congress Milan March 2023. https://uroweb.org/eau-guidelines/citing-usage-republication.

[19] Cash H., Slowinski T., Buechler A., Grimm A., Friedersdorff F., Schmidt D., Miller K., Giessing M., Fuller T.F. (2012). Impact of surgeon experience on complication rates and functional outcomes of 484 deceased donor renal transplants: A single-centre retrospective study. BJU Int..

[20] Nicolle L.E., Gupta K., Bradley S.F., Colgan R., DeMuri G.P., Drekonja D., Eckert L.O., Geerlings S.E., Köves B., Hooton T.M. (2019). Clinical Practice Guideline for the Management of Asymptomatic Bacteriuria: 2019 Update by the Infectious Diseases Society of America. Clin. Infect. Dis..

[21] Favi E., Spagnoletti G., Valentini A., Tondolo V., Nanni G., Citterio F., Castagneto M. (2009). Long-term clinical impact of vesicoureteral reflux in kidney transplantation. Transplant. Proc..

[22] Gołębiewska J., Dębska-Ślizień A., Komarnicka J., Samet A., Rutkowski B. (2011). Urinary tract infections in renal transplant recipients. Transplant. Proc..

[23] Pellé G., Vimont S., Levy P.P., Hertig A., Ouali N., Chassin C., Arlet G., Rondeau E., Vandewalle A. (2007). Acute pyelonephritis represents a risk factor impairing long-term kidney graft function. Am. J. Transplant..

[24] Kamath N., John G., Neelakantan N., Kirubakaran M., Jacob C. (2006). Acute graft pyelonephritis following renal transplantation. Transpl. Infect. Dis..

[25] Giral M., Pascuariello G., Karam G., Hourmant M., Cantarovich D., Dantal J., Blancho G., Coupel S., Josien R., Daguin P. (2002). Acute graft pyelonephritis and long-term kidney allograft outcome. Kidney Int..

[26] Coulthard M.G., Keir M.J. (2006). Reflux nephropathy in kidney transplants, demonstrated by dimercaptosuccinic acid scanning. Transplantation.

[27] Muller V., Becker G., Delfs M., Albrecht K.-H., Philipp T., Heemann U. (1998). Do urinary tract infections trigger chronic kidney transplant rejection in man?. J. Urol..

[28] Ranchin B., Chapuis F., Dawhara M., Canterino I., Hadj-Aïssa A., Saïd M., Parchoux B., Dubourg L., Pouillaude J., Floret D. (2000). Vesicoureteral reflux after kidney transplantation in children. Nephrol. Dial. Transplant..

[29] Krishnan A., Swana H., Mathias R., Baskin L.S. (2006). Redo ureteroneocystostomy using an extravesical approach in pediatric renal transplant patients with reflux: A retrospective analysis and description of technique. J. Urol..

[30] Duty B.D., Conlin M.J., Fuchs E.F., Barry J.M. (2013). The current role of endourologic management of renal transplantation complications. Adv. Urol..

